# Unique Type I Interferon Responses Determine the Functional Fate of Migratory Lung Dendritic Cells during Influenza Virus Infection

**DOI:** 10.1371/journal.ppat.1002345

**Published:** 2011-11-03

**Authors:** Bruno Moltedo, Wenjing Li, Jacob S. Yount, Thomas M. Moran

**Affiliations:** 1 Department of Microbiology, Mount Sinai School of Medicine, New York, New York, United States of America; 2 Immunology Institute, Mount Sinai School of Medicine, New York, New York, United States of America; McMaster University, Canada

## Abstract

Migratory lung dendritic cells (DCs) transport viral antigen from the lungs to the draining mediastinal lymph nodes (MLNs) during influenza virus infection to initiate the adaptive immune response. Two major migratory DC subsets, CD103^+^ DCs and CD11b^high^ DCs participate in this function and it is not clear if these antigen presenting cell (APC) populations become directly infected and if so whether their activity is influenced by the infection. In these experiments we show that both subpopulations can become infected and migrate to the draining MLN but a difference in their response to type I interferon (I-IFN) signaling dictates the capacity of the virus to replicate. CD103^+^ DCs allow the virus to replicate to significantly higher levels than do the CD11b^high^ DCs, and they release infectious virus in the MLNs and when cultured *ex-vivo*. Virus replication in CD11b^high^ DCs is inhibited by I-IFNs, since ablation of the I-IFN receptor (IFNAR) signaling permits virus to replicate vigorously and productively in this subset. Interestingly, CD103^+^ DCs are less sensitive to I-IFNs upregulating interferon-induced genes to a lesser extent than CD11b^high^ DCs. The attenuated IFNAR signaling by CD103^+^ DCs correlates with their described superior antigen presentation capacity for naïve CD8^+^ T cells when compared to CD11b^high^ DCs. Indeed ablation of IFNAR signaling equalizes the competency of the antigen presenting function for the two subpopulations. Thus, antigen presentation by lung DCs is proportional to virus replication and this is tightly constrained by I-IFN. The “interferon-resistant” CD103^+^ DCs may have evolved to ensure the presentation of viral antigens to T cells in I-IFN rich environments. Conversely, this trait may be exploitable by viral pathogens as a mechanism for systemic dissemination.

## Author Summary

Migratory lung dendritic cells (DCs) control the initiation of the adaptive immune responses to influenza virus by expanding virus-specific T cells in draining lymph nodes (MLNs) that will subsequently clear the pathogen from the respiratory tract. Here we demonstrate that both subsets of lung DCs, CD103^+^ DCs and CD11b^high^ DCs become infected by influenza virus *in vivo* and migrate to the MLNs, but only CD103^+^ DCs support productive virus replication. Enhanced virus replication in CD103^+^ DCs compared to CD11b^high^ DCs was responsible for their superior antigen presentation efficacy for naïve CD8^+^ T cells and originated from a difference in sensitivity of the two DC populations to type I interferon (I-IFN). These data show that in contrast to most other immune cell types, DCs can become productively infected with influenza virus and I-IFN operates as a master regulator controlling which DC subset will present antigen during a viral infection. A deeper understanding of basic innate and adaptive immune response mechanisms regulated by I-FN may lead to the development of cutting edge therapies and improve vaccine efficacy against influenza and other viruses.

## Introduction

Influenza virus replicates productively in the epithelial cells of the respiratory tract [1], [2]. In close contact to the infected epithelial cells lies a network of specialized antigen presenting cells (APCs) known as dendritic cells (DCs) [3], [4]. Two major subsets of lung DCs known as CD103^+^ DCs and CD11b^high^ DCs can be identified in the steady-state [5], [6], [7], [8]. Following influenza virus infection these cells migrate to the draining mediastinal lymph nodes (MLNs) loaded with viral antigens (Ag) [9], [10], [11], [12] to initiate T cell responses that are critical for virus clearance and recovery from infection [13], [14], [15]. The strategic localization of lung DCs adjacent to the productively infected epithelial cells ensures a supply of viral antigen for presentation to T cells, but also makes DCs an ideal target for virus infection.

Following aerosol infection of mice [9], [16], lung DCs begin to migrate 2 days post-infection (dpi) concomitant with the abrupt production of type I interferons (I-IFNs) and a myriad of other pro-inflammatory cytokines [10], [17]. I-IFNs have potent antiviral activity limiting virus replication in infected cells by inducing the transcription of hundreds of interferon-stimulated genes (ISGs) [18], [19], [20], [21]. The induction of ISGs or the antiviral state by I-IFNs, and other related cytokines such as interferon-lambda, also protect adjacent cells from infection thus restricting unabated spread of the virus in the respiratory tract [22], [23]. I-IFNs have also been shown to function as natural adjuvants for maturing human [24] and mouse DCs *in vitro* [25] and splenic DCs [26] *in vivo*. The timing of I-IFN production and lung DC migration are temporally related and it is not known to what extent I-IFNs influence the function of these cells and their interaction with influenza virus.

Here we investigated the role of I-IFN in lung DC function during influenza virus infection by studying the two major migratory lung DC subsets: CD103^+^ DCs and CD11b^high^ DCs. We demonstrate a striking dichotomy in the sensitivity to I-IFN by lung DCs that defines the level of virus replication and Ag presentation to virus-specific naïve CD8^+^ T cells. Additionally, our findings show evidence that CD103^+^ DCs permit productive influenza replication and release infectious virus in the draining lymph nodes while CD11b^high^ DCs do not.

## Results

### Influenza viral mRNA transcripts and infectious virus can be demonstrated in the draining MLNs only after 2 days post-infection

**Figure 1:**
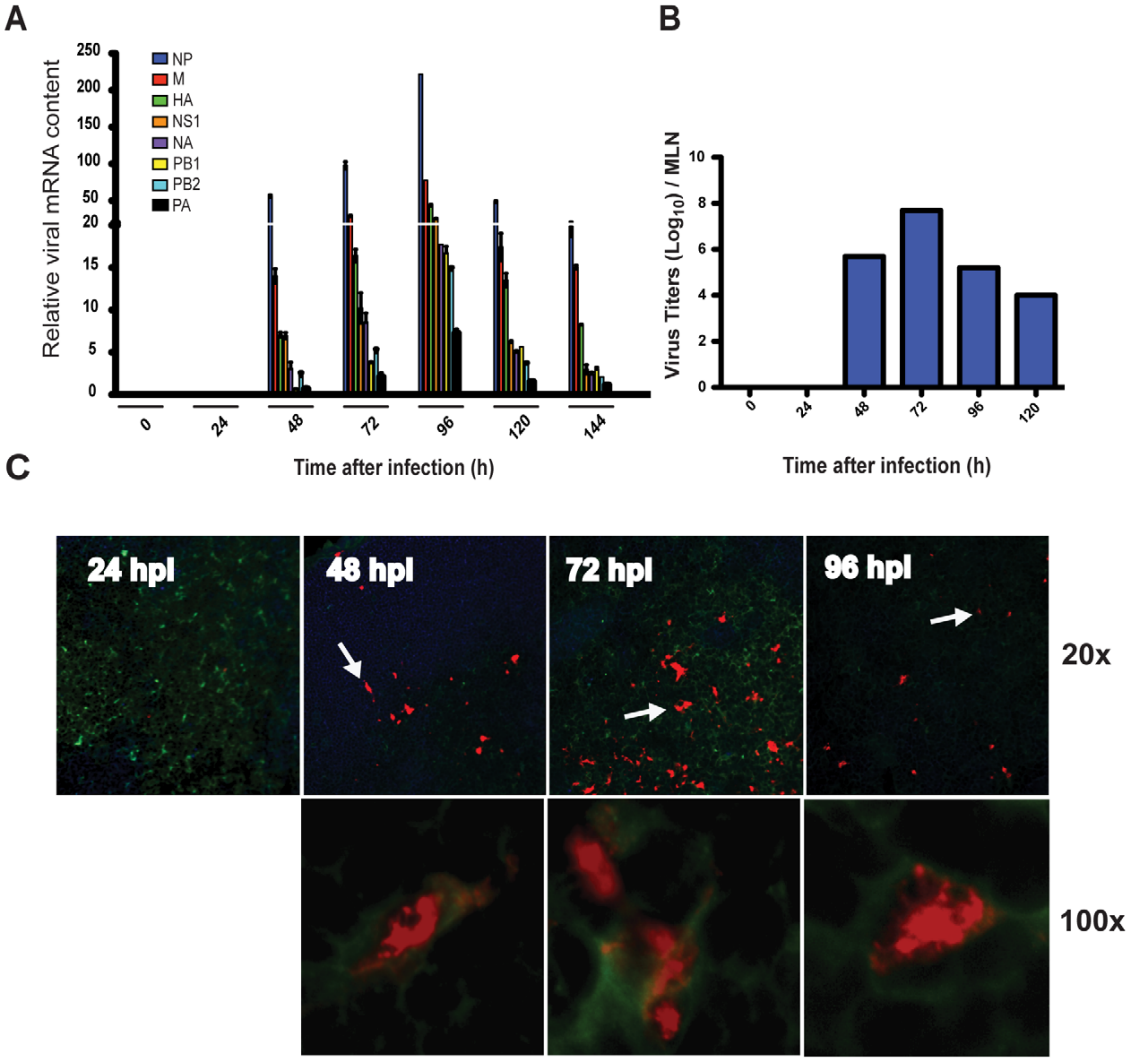
Ag-bearing DC migration is associated with localized viral mRNA and virus replication in the MLNs. A. Total MLN cDNA obtained from PR8-infected mice, at different time points after infection, was analyzed by qPCR for the 8 segments of influenza viral mRNA (NP (nucleoprotein), HA (hemagglutinin), M (matrix protein), NS1 (non-structural protein 1), NA (neuraminidase), PB1 (polymerase PB1), PB2 (polymerase PB2), and PA (polymerase PA) B. MLNs were isolated from PR8-infected mice and titers of infectious virus were determined. C. Cryostat sections of MLNs isolated from PR8-infected mice were fixed, permeabilized and stained for CD11c (green), NP, M (red) and B220 (blue), and visualized with an immunofluorescence microscope at 20x and 100x magnification. White arrows indicate Ag-bearing CD11c^+^ cells at 20x magnification that were then zoomed and photographed at 100x.

Our previous work showed that influenza virus suppressed innate immunity in the lungs of mice for the first 2 days after infection; a period we termed “the stealth phase” [10], [27]. During this privileged time, virus replication proceeds without instigating inflammation or triggering the migration of lung DCs to the MLNs. The end of the stealth phase is characterized by the abrupt and vigorous production of I-IFNs and cytokines and the initiation of Ag-bearing DC migration to the MLNs [10], [28]. The cells arriving in the MLNs were identified by the presence of intracellular viral proteins and no determination was made as to whether they were actually infected or simply carrying viral proteins from the site of infection. To distinguish these two possibilities we isolated MLNs from PR8-infected mice at defined time points after aerosol infection and measured viral mRNA transcripts from all 8 segments of influenza virus. As shown in Figure 1A transcripts of all 8 segments were identified in the MLN of infected mice. The kinetics of the appearance of the viral mRNA in the MLN correlated perfectly with our previous results for Ag-bearing DC migration [10] as it was only detectable starting at 48 hpi, increased steadily, peaked by 96 hpi, and persisted for at least 6 dpi. Homogenates collected from the MLN demonstrated the presence of infectious virus particles at time points that correlated with the appearance of viral mRNA and Ag-bearing DC migration (Figure 1B) [9], [10], [11]. Inspection of CD11c^+^ cells in the MLNs by immunohistochemistry confirmed viral antigen positive cells only after 48 hpi (Figure 1C).

### Viral transcripts are carried to the MLNs by migratory cells

**Figure 2:**
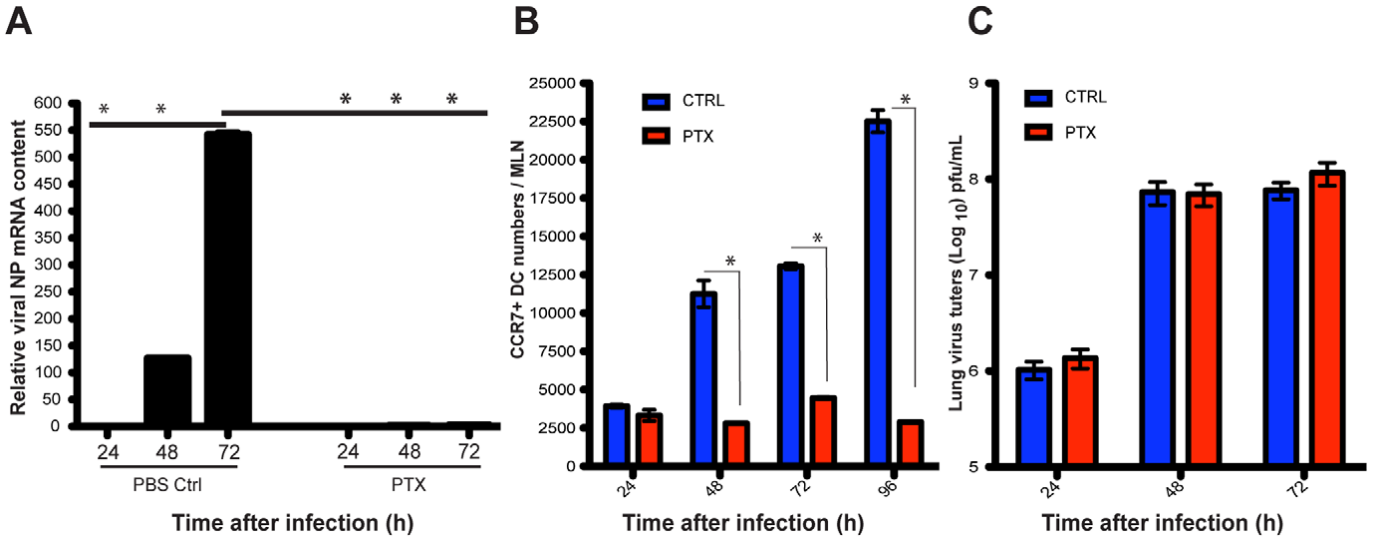
Blockade of lung migratory cells abolishes viral mRNA production in the MLNs. A. Two hours after infection, mice were challenged intranasally with a single dose of pertussis toxin (PTX) or PBS (Ctrl), and thereafter a daily single dose of PTX or PBS was given i.p to each mouse. Total RNA of MLNs was isolated at different time points after infection and viral mRNA (NP segment) was quantitated by qPCR. B. Absolute numbers of CCR7^+^ DCs in the MLNs during A/PR8 infection of mice were measured. Blue bars represent PBS mock treated mice and red bars mice treated with PTX. C. Lung virus titers of influenza infected mice either challenged with PTX (red bars) or mock challenged with PBS (blue bars). Error bars represent mean +/− SD, * p<0.05.

Migration of lung DCs is controlled by the chemokine receptor CCR7 [8], [29], [30], and can be blocked by the use of pertussis toxin (PTX) [7]. Intranasal PTX administration to infected mice completely abolished the appearance of viral mRNA in the MLNs (Figure 2A), and significantly reduced the numbers of CCR7^+^ DCs in the MLNs (Figure 2B) but had no effect on virus replication in the lungs (Figure 2C). These data show that virus reaches the draining MLNs by cell-associated transport rather than by leakage of free virus through efferent lymphatics.

### Infectious virus is carried by lung migratory DCs

**Figure 3:**
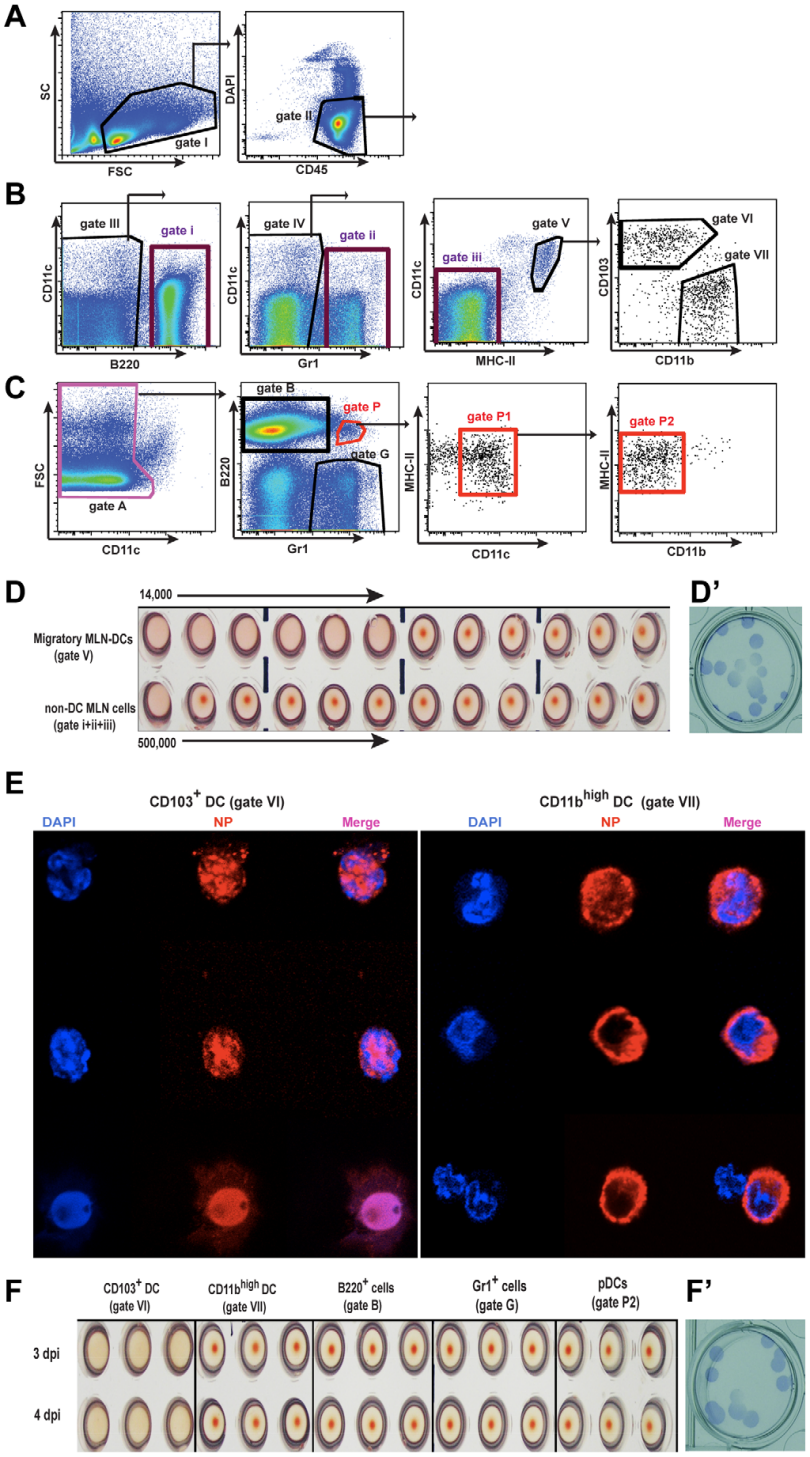
Migratory CD103^+^ DCs are the major cell type carrying infectious virus particles to the MLNs. A–C. Isolation of different MLN cell populations from influenza infected mice by cell sorting. A. Live gate for total MLN leukocytes shown as CD45^+^ DAPI^-^ cells (gate II). B. Sorting strategy to obtain total migratory DCs (gate V), individual CD103^+^ DCs (gate VI), or CD11b^high^ DCs (gate VII), and non-DC MLN cells by pooling gates i, ii, and iii during collection. All these cell populations were selected from the total CD45^+^ live cell gate I. C. Alternative sorting strategy starting with gate II, to obtain B220^+^ cells (gate B), Gr1^+^ cells (gate G), and pDCs (gate P2). D. Total migratory DCs (gate V) comprised of CD103^+^ DCs and CD11b^high^ DCs, or MLN non-DC cells (gates I, ii, and iii pooled) sorted at 3 dpi, were layered over MDCK cells in a 1∶2 serial dilution, starting with 14,000 and 500,000 cells respectively, and were co-cultured in the presence of TPCK-trypsin for 3 days. Supernatants were assayed for infectious virus particles by hemagglutination of RBCs. D'. Supernatants from MLN-DC/MDCK co-cultures were assayed for infectious virus by plaque immunostaining on MDCK cells. E. Immunofluorescence of intracellular viral NP of sorted CD103^+^DCs and CD11b^high^ DCs from infected mice at 3 dpi. F. Individual cell populations, isolated by sorting at day 3 and 4 post-infection, as described above, were injected into 10-day old embryonated eggs, and 2 days later, allantoic fluid was assayed for infectious virus particles by hemagglutination of RBCs. Each cell type at each time point was assayed in triplicate. F’. Allantoic fluid from eggs injected with CD103^+^ DCs was assayed for infectious virus particles by a plaque immunostaining assay on MDCK cells.

Both of the major lung DC subsets, CD103^+^ DCs and CD11b^high^ DCs, transport viral Ag to the MLNs [11], [31], but whether it is in the form of infectious virus has not been determined. Figure 3A-C shows a sorting strategy that was used to isolate migratory DCs and other cells from the MLNs of infected animals. Total migratory DCs were isolated and co-cultured (gate V, Figure 3B) with virus permissive MDCK cells *in vitro* using decreasing numbers of cells. The DC-depleted lymph node cells were similarly cultured with MDCK cells (Figure 3B, gate i-iii pooled together). Infectious virus was isolated from MDCK cells cultured with 1,000 fold less migratory DCs than was observed when DC-depleted lymph node cells were used indicating that DCs were the primary transporters of infectious virus to the MLNs (Figure 3D). Plaque immunostaining of MDCKs infected with supernatant from these co-cultures confirmed the presence of live virus (Figure 3D’).

### CD103^+^ DCs carry infectious virus from the lungs to MLNs during infection

**Figure 4:**
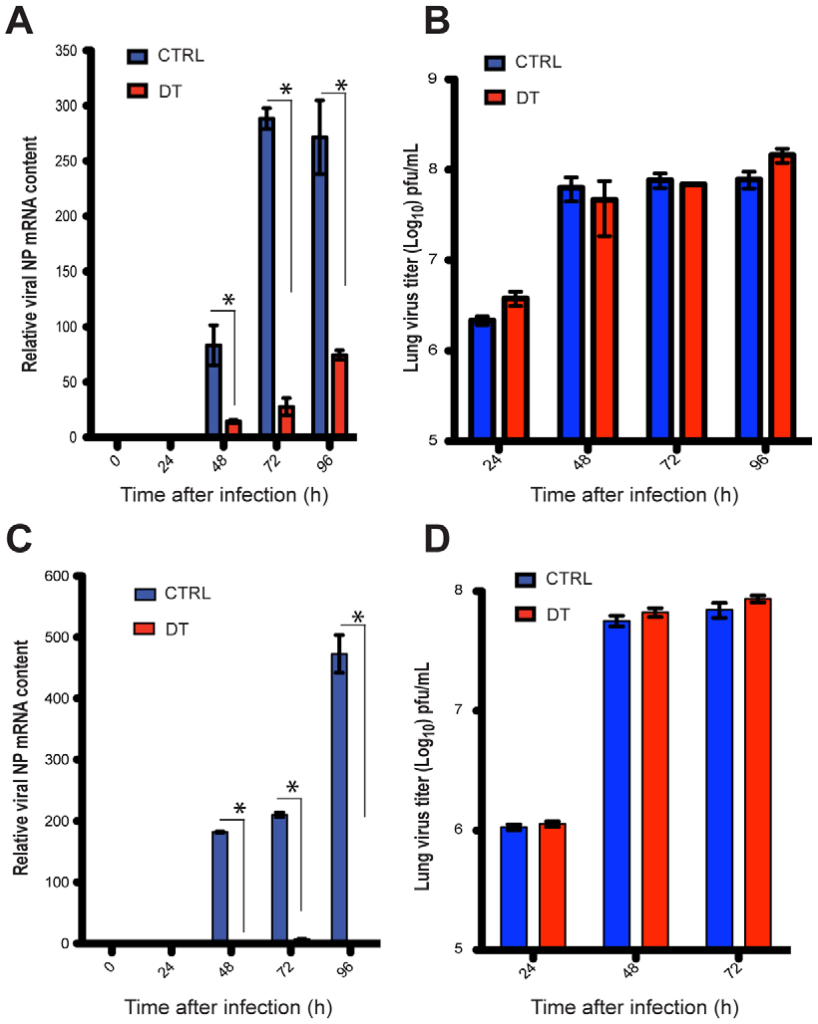
Depletion of total DCs or Langerin^+^ DCs compromises viral load in the MLNs during infection. A. Langerin-DTR-EGFP mice were injected i.p with DT daily, starting one day before infection with PR8. MLNs were harvested and NP mRNA was quantitated by qPCR. B. Lung virus titers of DT-treated PR8 infected Langerin-DTR-EGFP mice (red bars) or mock treated (blue bars). C and D. CD11c-DTR-EGFP mice were used instead, performing the same experiments as described in A and B. Error bars represent mean +/- SD, * p<0.05.

When individual migratory lung DC subsets were stained for viral NP and visualized by confocal microscopy both CD103^+^ DCs (gate VI, Figure 3B) and CD11b^high^ DCs (gate VII, Figure 3B) were found to have abundant intracellular Ag (Figure 3E). NP co-localized to the nucleus in CD103^+^ DCs (Figure 3E). In contrast, NP in CD11b^high^ DCs surrounded but did not co-localize with the nucleus (Figure 3E). To test which DC subset transferred infectious virus particles to MDCKs *in vitro*, CD103^+^ DCs and CD11b^high^ DCs isolated from MLNs at 72 or 96 hpi were injected separately into embryonated-chicken eggs (Figure 3F). 40 h later the allantoic fluid was collected and tested for the presence of replicating virus by agglutination of chicken red blood cells (RBCs). Only CD103^+^ DCs (gate VI, Figure 3B) injected into eggs led to the production of virus particles (Figure 3F), and the presence of infectious virus was confirmed by plaque immunostaining of MDCKs infected with the allantoic fluid from these samples (Figure 3F’). Neither CD11b^high^ DCs (gate VII, Figure 3B) nor any other major leukocyte populations (gate B: B220^+^ cells; gate G: Gr1^+^ cells; gate P2: pDCs, Figure 3C) caused infectious virus production in embryonated eggs (Figure 3F). Moreover, depletion of Langerin^+^ CD103^+^ DCs in PR8-infected Langerin-DTR EGFP mice [32] reduced virus mRNA significantly in the MLNs (Figure 4A), with no apparent compromise of lung virus titers (Figure 4B). Total depletion of CD11c^+^ cells from infected mice virtually eliminated viral mRNA from the MLN [33] (Figure 4C and 4D).

Multicycle replication of PR8 virus *in vitro* requires L-1-tosylamido-2-phenylethyl chloromethyl ketone treated-trypsin (TPCK-trypsin) to promote HA cleavage and spread to uninfected cells [34]. We next tested whether virus infection of MDCKs via contact with migratory DCs was dependent on TPCK-trypsin. As shown in Figure S1A, MDCK cells were infected in the absence of trypsin when co-cultured for 2 days with migratory DCs (see black arrows), showing that the transfer of infectious virus to MDCKs was independent of an exogenous added protease. As expected, subsequent robust spread of PR8 virus in MDCK cells was dependent on TPCK-trypsin (Figure S1A). We repeated the experiment with the closely related influenza strain known as WSN virus that is not dependent on TPCK-trypsin for multicycle replication [35], [36]. MDCKs co-cultured with MLN-DCs sorted from WSN infected mice were infected independently of trypsin (Figure S1A). Similar to the ability of CD103^+^ DCs to transfer infectious virus to embryonated eggs (Figure 3), virus could be transferred to MDCK cells upon co-culture with particular DC subsets isolated from PR8 and WSN infected mice. Specifically, only CD103^+^ DC but not CD11b^high^ DCs caused *ex-vivo* virus infection of MDCKs, and trypsin was necessary for multicycle replication depending on the type of virus utilized (Figure S1B).

### CD103^+^ DCs have an attenuated IFNAR signaling response that results in enhanced virus replication

As shown here and in previous work, at 2 dpi a rapid inflammatory response develops in the infected lung creating a milieu of proinflammatory cytokines including I-IFNs. IFNAR signaling in hematopoietic cells such as DCs induces the expression of several ISGs that promote the antiviral state [37], [38], [39]. The migration of lung DCs is initiated amidst this inflammatory environment and it was surprising that viral replication in the CD103^+^ DCs was not inhibited by the antiviral response triggered by I-IFN signaling [10], [40].

**Figure 5:**
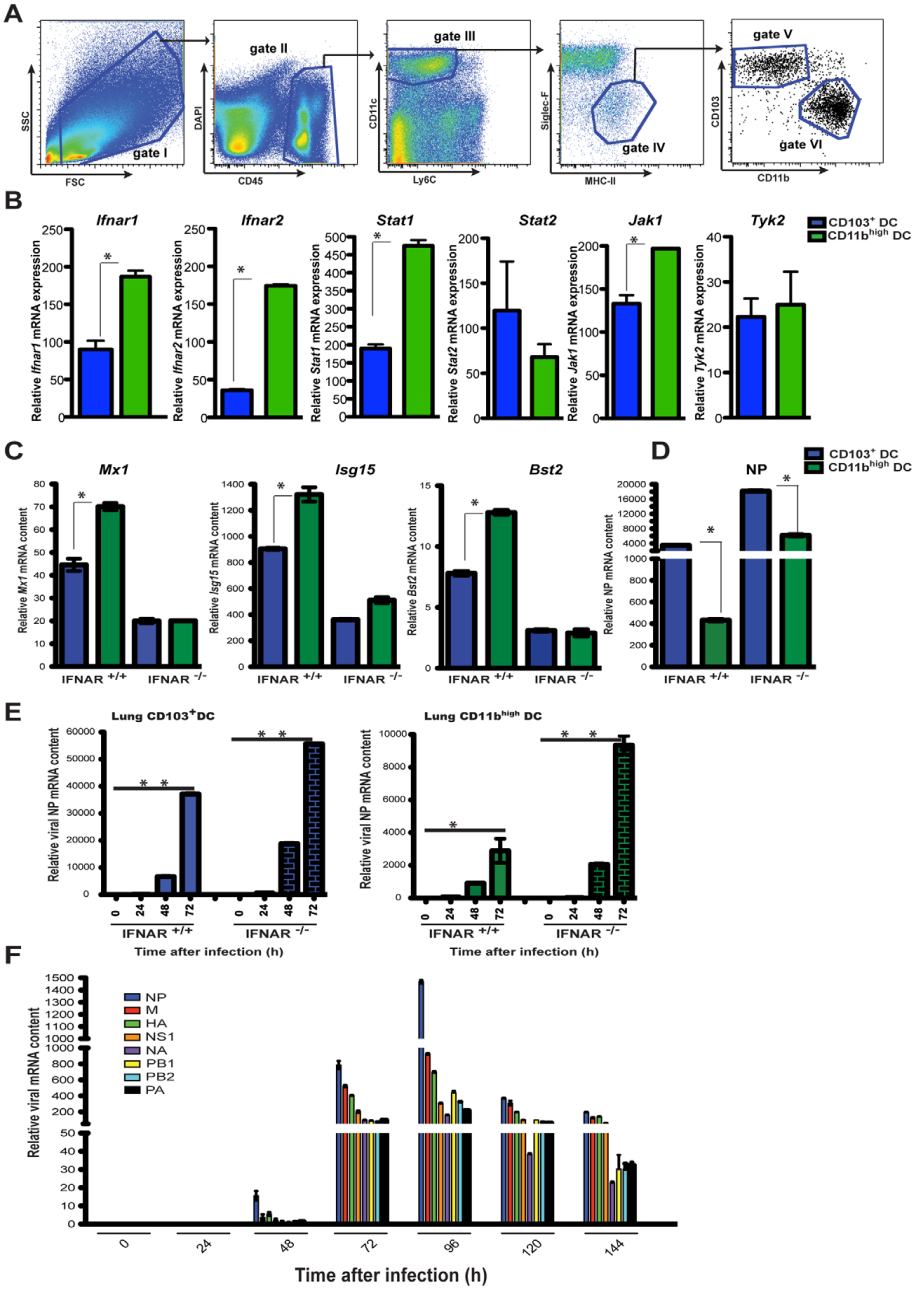
Unique responses to I-IFNs by lung DC subsets determine virus replication. A. Sorting strategy for lung DCs. Lung DCs are defined as CD45^+^ CD11c^+^ MHC-II^+^ Siglec-F^-^ Ly6C^-^ cells (gates I-IV). From gate IV individual DC subsets were further gated into CD103^+^ DC (gate V) and CD11b^high^ DC (gate VI) for separation by cell sorting. B. qPCR analysis of *Ifnar* genes and signaling related genes in lung CD103^+^DCs (blue bars) and lung CD11b^high^ DCs isolated from IFNAR ^+/+^ naïve mice. C. qPCR analysis of ISG expression by MLN CD103^+^ DCs (blue bars) or MLN CD11b^high^ DCs (green bars) sorted from either IFNAR ^-/-^ or IFNAR ^+/+^ PR8-infected mice at 4 dpi. D. Expression of viral NP mRNA was determined by qPCR for individual sorted MLN DC subsets as in (C). E. CD103^+^ DCs and CD11b^high^ DCs isolated from the lungs of IFNAR ^-/-^ or IFNAR ^+/+^ mice during the course of PR8 infection were assayed for viral NP mRNA by qPCR. F. Total MLN cDNA obtained from PR8-infected IFNAR ^-/-^ mice, at different time points after infection, was analyzed for mRNA of the 8 segments of influenza virus (NP, HA, M, NS1, NA, PB1, PB2, PA) by qPCR. Error bars represent mean +/− SD, * p<0.05.

Therefore we compared elements associated with IFNAR signaling in steady state CD103^+^ DCs and CD11b^high^ DCs sorted from lungs of naïve mice (sorting strategy, Figure 5A). As shown in Figure 5B, lung CD103^+^ DCs show a reduced expression of *Ifnar1* and *Ifnar2* chains, the *Jak1* kinase, as well as the transcription factor *Stat1,* when compared to lung CD11b^high^ DCs. These four genes represent key elements of the IFNAR receptor-signaling complex. To determine if the decreased expression of IFNAR signaling components translates into differences in ISG upregulation CD103^+^ DCs and CD11b^high^ DCs were sorted from the MLNs during infection. Both DC subsets found in the MLNs of infected animals showed upregulated ISGs, however wild type CD11b^high^ DCs produced higher levels of the ISG gene products than CD103^+^ DCs, a difference that was abrogated in the absence of IFNAR signaling (Figure 5C). In agreement with a differential IFNAR signaling and ISG response by lung DCs, CD103^+^ DCs collected from MLNs of infected animals showed greater permissivity to virus replication when compared to CD11b^high^ DCs, expressing approximately ten times more viral NP mRNA (Figure 5D). IFNAR deficiency exacerbated the virus burden in CD103^+^ DCs and increased significantly the viral mRNA content in CD11b^high^ DCs (Figure 5D). The difference in virus susceptibility between the CD103^+^ DCs and CD11b^high^ DCs can be observed at the earliest time points in DCs isolated directly from the lungs of infected mice and mirrors the observations in cells collected from the MLN (Figure 5E). These results suggest that IFNAR signaling effectively restricts virus replication in CD11b^high^ DCs whereas CD103^+^ DCs are somewhat refractory to this cytokine. Consistent with these findings, viral mRNA levels in the MLN of infected IFNAR ^-/-^ mice were significantly higher than in wild type animals (Figure 1A) indicating that the amount of virus transported to the MLN was higher in IFNAR ^-/-^ mice (Figure 5F).

### IFNAR signaling determines infection and the division of labor among lung DCs during influenza virus infection

**Figure 6:**
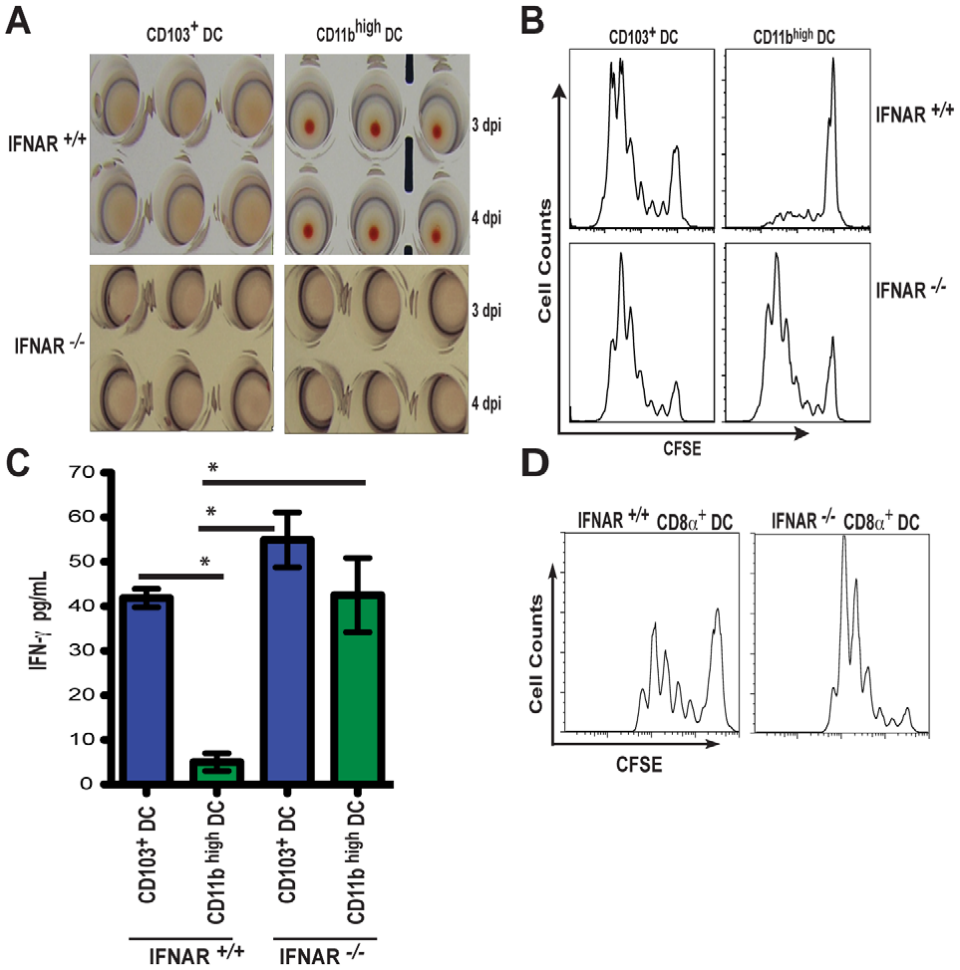
IFNAR signaling determines class-I restricted antigen presentation by CD11b ^high^ DCs during influenza virus infection. A. Individual MLN DC subsets from IFNAR ^+/+^ or IFNAR ^-/-^ mice were isolated by cell sorting at 3 and 4 dpi (described in Figure 3), and injected into 10-day embryonated eggs. 2 days later, allantoic fluid was assayed for infectious virus particles by hemagglutination of RBCs. B. Sorted MLN CD103^+^ DCs and CD11b^high^ DCs from either IFNAR ^+/+^ or IFNAR ^-/-^ mice infected with PR8-OT-I were isolated at 4 dpi and co-cultured for 60 h with CFSE-labeled naïve OT-I CD8^+^ T cells. T cell proliferation was analyzed by flow cytometry. C. IFN-γ production of proliferating OT-I cultures (B) were assayed by ELISA. D. Lymph-node resident CD8α^+^ DCs isolated at 4 dpi from PR8-OT-I-infected IFNAR ^+/+^ or IFNAR ^-/-^ mice were co-cultured with naïve OT-I CD8^+^ T cells and proliferation was assessed by CFSE dilution. Error bars represent mean +/- SD, * p<0.05.

To determine if the increase in viral mRNA seen in the MLNs from IFNAR ^-/-^ mice could result from enhanced virus replication from migratory lung DCs, we sorted CD103^+^ DCs and CD11b^high^ DCs from infected IFNAR ^-/-^ and IFNAR ^+/+^ mice at 3 and 4 dpi. Individual DC subsets were injected into embryonated eggs and the presence of infectious virus determined 40 h later by hemagglutination of the allantoic fluid. In contrast to what was observed with CD11b^high^ DCs from wild type animals, IFNAR ^-/-^ CD11b^high^ DCs were now permissive for productive infection by influenza virus (Figure 6A).

Data from a number of laboratories have confirmed that CD103^+^ DCs are superior APCs for the priming of naïve CD8^+^ T cells [11], [12], [31]. Our data indicated a possible relationship between virus replication and potent Ag presentation capacity. Therefore we isolated CD103^+^ DCs and CD11b^high^ DCs from IFNAR ^+/+^ and IFNAR ^-/-^ mice infected with PR8-OT-I virus to determine whether suppressed viral replication in CD11b^high^ DCs by IFNAR signaling would be the etiological reason behind this difference in Ag presentation.

As anticipated, *in vitro* proliferation of naïve CD8^+^ OT-I T cells was superior when co-cultured with IFNAR ^+/+^ CD103^+^ DCs compared to IFNAR ^+/+^ CD11b^high^ DCs (Figure 6B). Strikingly, OT-I T cell proliferation was comparable when both DC subsets were isolated from IFNAR ^-/-^ mice (Figure 6B) and the production of interferon-gamma (IFN-γ) from the OT-I T cells was almost equivalent (Figure 6C). As a control, lymph-node resident CD8α^+^ DCs, known to be potent APCs for CTL priming [12], were sorted from PR8-OT-I infected IFNAR ^+/+^ and IFNAR ^-/-^ mice (sorting strategy, Figure S2) and co-cultured with OT-I cells *in vitro* (Figure 6D). IFNAR ^+/+^ CD8α^+^ DCs induced OT-I T cell proliferation in vitro but not as robustly as IFNAR ^+/+^ CD103^+^ DC. In contrast, IFNAR ^-/-^ CD8α^+^ DCs gained an enhanced ability to present Ag in the absence of IFNAR signaling similarly to what is observed for CD11b^high^ DCs. These data show that viral replication in a given migratory DC population correlates with priming efficiency of naïve CD8^+^ T cells and the observed differences in priming are not necessarily an intrinsic feature of each DC subset and can be modulated by I-IFN signaling.

## Discussion

The lung serves as a portal for the entry of a myriad of respiratory viruses throughout the lifetime of the host. Induction of cellular T cell immunity requires the obligate interaction of lung DCs with viruses, with the imminent risk of infection. Our data demonstrates that both lung DC subsets are infected with influenza virus *in vivo* and this event does not impair their migration to the MLNs or their function as APCs. By carefully examining each lung DC subset we found that CD103^+^ DCs had much greater levels of viral mRNA and produced infectious virus particles, a finding that challenges the widely held belief that influenza virus replicates only in the lung epithelium [1], [2]. On the other hand, CD11b^high^ DCs expressed viral mRNA to a much lesser extent and did not support virus growth *ex-vivo* suggesting that these cells were abortively infected. The dramatic differences in virus susceptibility were striking and led us to investigate whether I-IFNs were uniquely sensed by both DC subsets.

Based on our previous observations, DCs begin to migrate *in vivo* only when lung inflammation is triggered ∼2 dpi with influenza virus [10] [27]. At this time, the antiviral cytokines, I-IFNs, are readily abundant but their effects on lung DC function were not previously addressed. Importantly, I-IFNs confer only partial protection to leukocytes during respiratory virus infection *in vivo* [40], and this is further supported by *in vitro* experiments where DCs and other leukocytes sustain some degree of viral transcription even after treatment with very high concentrations of I-IFNs [41], [42]. Therefore, migrating lung DCs are not necessarily refractory to influenza virus infection, even if they are I-IFN-primed before encountering the virus. In the present study we found that both lung DCs displayed an “interferon signature” (upregulation of ISGs) but the magnitude of the responses was quite different depending on the DC subset analyzed. Indeed, CD103^+^ DCs were found to be “inherently resistant” to IFNAR signaling with an attenuated ISG response when compared to CD11b^high^ DCs. Consequently, our confocal imaging data showed nuclear localization of NP only in CD103^+^ DCs but not in CD11b^high^ DCs suggesting that the latter subset has a tight control over virus replication mediated by stronger I-IFN signaling.

By ablating IFNAR signaling in CD11b^high^ DCs we could increase their viral mRNA replication to levels comparable to wild type CD103^+^ DCs, and it was enough to promote infectious virus production by CD11b^high^ DCs. Experimental evidence suggests a divergent hematopoetic origin of lung DC subsets. CD103^+^ DCs appear to arise from pre-DC precursors while CD11b^high^ DCs are monocyte derived [43], [44], [45], [46], and this dichotomy could determine unique IFNAR signaling differences. Currently we are studying in more detail the underlying mechanisms explaining why CD103^+^ DCs have an attenuated IFNAR signaling and an inherent susceptibility to virus infection. Some of these include, inhibitory genes downstream of the IFNAR signaling pathway [47], [48], silencing of ISGs by epigenetic modifications of chromatin [49], specific expression of microRNAs that favor virus replication or targeting of particular IFNAR signaling components [50], and signaling by suppressor cytokines such as IL-10 [51].

The differential susceptibilities to virus infection by both lung DCs suggested that the relative viral Ag abundance might act as a limiting factor for peptide presentation by mayor histocompatibility class-I (MHC-I) molecules. CD103^+^ DCs have been characterized as APCs with superior abilities to prime naïve CD8^+^ T cells relative to CD11b^high^ DCs, though both are equally competent at priming naïve CD4^+^ T cells [11], [31], [52]. The underlying reason for this fundamental difference has been ascribed to a division of labor theory [53](first described for splenic DCs) where unique gene programs define specialized machinery for MHC-II or MHC-I Ag presentation within each DC subset [8]. Here we propose an alternative model for this dichotomy based on the strength of their response to I-IFNs that determines the efficiency in MHC-I Ag presentation by controlling the level of virus replication within each DC subset. The prominent natural susceptibility of CD103^+^ DCs to virus infection may represent a mechanism that ensures adequate MHC-I Ag presentation in a cytokine milieu dominated by I-IFNs [10], [40], [42] leading to a higher density of peptide-MHC-I complexes on the cell surface facilitated by constant degradation of newly synthesized viral proteins [54], [55], [56], [57]. On the other hand, decreased virus replication in CD11b^high^ DCs reduces peptide-MHC-I complex production, but loss of IFNAR signaling boosts viral replication and their ability to prime naïve CD8^+^ T cells to levels comparable to CD103^+^ DCs.

In this study, we did not address whether cross-priming [58] represents a major component regulating Ag presentation by CD103^+^ DCs and CD11b^high^ DCs and further studies will be required to address their relationship to direct infection of lung DCs. Intriguingly, lymph node resident-CD8α^+^ DCs known to be potent APCs for cytotoxic T cells [12] were also boosted by the absence of IFNAR signaling. CD8α^+^ DCs might benefit from increased numbers of virus-infected DCs (CD103^+^ DC and CD11b^high^ DC) in infected IFNAR ^-/-^ mice that may undergo cell death or release viral Ag that upon endocytosis may be used as substrates for crosspriming. Alternatively, I-IFN signaling might be responsible for lymph-node CD8α^+^ DC crosspresentation in influenza-infected wild-type mice ensuring optimal DC maturation [26] and increased processing of viral antigen from migratory DCs [12]; despite tight control of virus replication by I-IFN.

The hypersensitive IFNAR signaling by CD11b^high^ DCs may divert these cells from naïve CTL priming to accomplish other specific tasks in the immune response to influenza virus, which is essentially multifactorial [15], [59]. Some of these functions may include, the production of high affinity IgG antibodies that may depend upon the continuous migration of antigen-bearing CD11b^high^ DCs derived from inflammatory monocytes [60], [61], [62], restimulation of effector CD4^+^ and CD8^+^ T cells locally in the lungs to trigger cytokine production and cytotoxicity [63], and as potent cytokine and chemokine secretion machines activating and recruiting innate and adaptive effector cell populations to the lungs and lymph nodes [64]. Additionally, the robust I-IFN response may have a protective role in sparing CD11b^high^ DCs from death that may explain late antigen presentation during infection to CTLs in the MLNs and lungs, which lingers long after the virus has been cleared [65], [66], [67], [68]. This knowledge about CD11b^high^ DCs can potentially be harnessed to improve live DC vaccines in human medicine. The likely human version of CD11b^high^ DCs is easily derived from blood monocytes [69], [70] and is commonly used for *in vivo* immunization protocols [71]. IFNAR signaling suppression in these cells may increase the replication of recombinant attenuated viruses or vectors improving vaccine efficacy for diverse diseases such as cancer [72], [73], [74] and HIV [75], [76], [77], [78].

**Figure 7:**
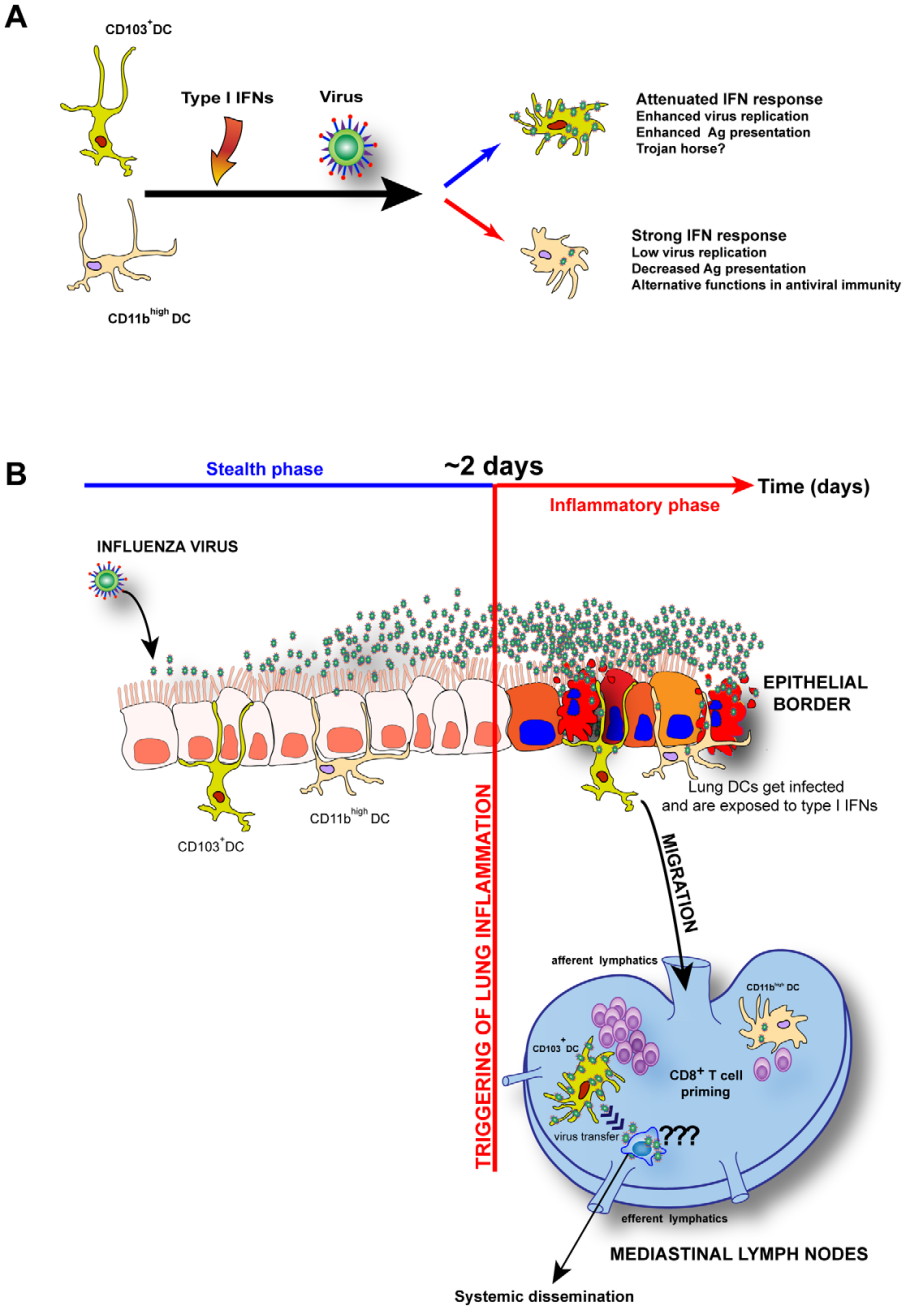
Model: Unique I-IFNs responses determine the functional fate of migratory lung DCs during respiratory virus infection. A. CD103^+^ DCs are naturally resistant to IFNAR signaling and allow virus replication to higher levels leading to enhanced antigen presentation, when compared to CD11b^high^ DCs that respond strongly to I-IFNs and impede virus replication and Ag presentation. B. The *in vivo* implications of virus infection and unique IFNAR signaling by CD103^+^ DCs and CD11b^high^ DCs during influenza virus infection and other respiratory viruses. Both lung DCs begin to migrate to the MLNs when lung inflammation is initiated around 2 days post-infection, an event that is preceded by unabated virus replication without an apparent innate response (stealth phase). IFNAR signaling is not protective in CD103^+^ DCs and their inherent susceptibility to virus infection may make them function as “Trojan horses” that transfer virus to other leukocytes in the MLNs and upon exit through efferent lymphatics disseminate virus systemically.

A danger inherent to the unique sensitivity of CD103^+^ DCs to infection is that viruses likely evolved to exploit these cells as shuttles and spread to multiple locations. Various viruses of worldwide health concern, such as measles and varicella zoster use the respiratory tract as an entry route from where they disseminate systemically [79], [80]. It would not be surprising, if these viruses hijacked the CD103^+^ DC equivalent in the human lung as an initial Trojan horse, and upon arrival to the MLNs spread to other susceptible cells that travel out of the lymph nodes into systemic circulation. The same might be happening with particular influenza virus strains in the human respiratory tract such as the highly pathogenic H5N1 virus whose HA contains a polybasic multi-cleavage site [81], and may target CD103^+^ DCs to spread hematogenously. Our experiments with WSN strain partially demonstrate this phenomenon, since virus could spread from CD103^+^ DCs *ex-vivo* and infect MDCKs without the aid of an exogenously added protease such as trypsin. It remains to be determined whether *in vivo*, CD103^+^ DCs can promote systemic spread of viruses and whether lymph-node specific proteases aid the cleavage of diverse hemagglutinins of influenza viruses. Altogether, our findings are presented as a model summarized in Figure 7, where the unique sensitivities to IFNAR signaling by individual lung DC subsets have divergent consequences for the adaptive immune response and as well as for viral pathogenesis.

## Materials and Methods

### Ethics statement

All animal work was conducted in agreement with approved protocols by the Institutional Animal Care and Use Committee (IACUC) at the Mount Sinai School of Medicine (Protocol Numbers #: 96-308 and 08-0951) and in accordance with guidelines in the Guide for the Care and Use of Laboratory Animals of the National Institutes of Health. The program is fully accredited by the Association for Assessment & Accreditation of Laboratory Animal Care, International (AAALAC).

### Mice and viruses

C57BL/6 wild type mice were purchased from Taconic or Jackson laboratories. OT-I transgenic mice [82] were purchased from Jackson Laboratories. OT-I mice were crossed to B6.SJL mice to generate CD45.1^+^ OT-I mice. C57BL/6 IFNAR ^-/-^ mice [83], [84] were kindly provided by Dr. Wilson (Immunology Department, University of Washington, USA). C57BL/6 Langerin diphtheria toxin receptor (DTR) EGFP [32] mice were kindly provided by Dr. Bernard Malissen (INSERM, Lyon, France). C57BL/6 CD11c-DTR EGFP mice [33] were purchased from Jackson laboratories. All mouse colonies were kept under pathogen-free conditions at the Animal Facility of the Mount Sinai School of Medicine.

Influenza virus strains A/PR/8/1934 (H1N1) (PR8), recombinant PR8-OTI, and A/WSN/1933 (H1N1) (WSN) were grown in 10-day embryonated eggs (Charles Rivers, Spafas). PR8-OT-I virus [85] was kindly provided by Dr. Peter Doherty and Dr. Paul Thomas (St. Jude's Research Children's Hospital, Memphis, TN).

### Mouse infection

Mice were infected using an Inhalation Exposure System A42X (Glass-Col, USA). Influenza virus was diluted in PBS to obtain a solution of 10^7.9^ virus particles/12 ml. The virus solution was placed inside of a sterile glass nebulizer connected to the infection chamber. Total exposure time with aerosolized virus was 30 min. Under these conditions 100% of animals were infected and showed reproducible lung titers in several experiments at every time point analyzed as described previously [9], [10].

WSN infection of mice was performed by intranasal infection. Briefly, mice were anesthetized with Avertin (Tribromoethanol, Acros Organics) by intraperitoneal injection, and 1,000 plaque forming units (pfu) of WSN virus in 35 µl PBS was applied intranasally to each mouse. Mice were monitored until they were fully awake and placed back into their cages.

### Lymph node and lung cell preparations for flow cytometry and fluorescence activated cell sorting (FACs)

MLNs from infected animals were mechanically dissociated and digested in DMEM/1% FCS supplemented with 0.25 mg/ml collagenase (Liberase type III, Roche) for 20 min at 37°C. Collagenase was neutralized by adding sterile PBS containing 2% FCS and 2.5 mM EDTA for 5 min, and then passing the dissociated cell suspension through a 70 µm strainer (BD Biosciences). Single cell suspensions were treated with red-blood cell lysis buffer (BD Biosciences) and resuspended in blocking buffer (PBS containing 2% FCS and 10 µg/ml Fc-receptor block). Lungs from infected mice were perfused with PBS to eliminate excess blood. Lung lobes were gently dissociated using forceps and digested for 50 min at 37°C in DMEM/1% FCS supplemented with collagenase-D (Roche) at 0.25 mg/ml. Single cell suspensions were obtained as described for MLNs.

For DC subset enumeration in the MLNs, lymph node cell suspensions were stained with antibodies against multiple surface antigens: anti-CD11c (clone HL-3), MHC-II (clone 2G9), Gr1 (Ly6C/Ly6G clone RBC865), Ly6C (Al-21), CD103 (clone 2E7), CD11b (clone M1/70), B220 (clone RA3-6B2), CD8α (clone 53-6.7). All antibodies were purchased from BD Bioscience, eBiosciences, and Biolegend. Lymph node DCs were identified as CD11c^high^, MHC-II ^high^, B220 negative, and Gr1 negative cells. Individual DC subsets were further gated as CD103^+^ DC and CD11b^high^ DC that were negative for CD8α. Lymph node resident CD8α^+^ DCs were identified in the MLN by gating on CD11c^high^ MHC-II ^intermediate^ cells as described elsewhere [86]. For enumeration of lung DC subsets, cell suspensions were stained with antibodies against multiple surface antigens: anti-CD11c (clone HL-3), MHC-II (clone 2G9), Gr1 (Ly6C/Ly6G clone RBC865), Siglec-F (clone E50-2440), CD103 (clone 2E7), CD11b (clone M1/70), B220 (clone RA3-6B2), CD8α (clone 53-6.7). Lung DCs were identified as CD11c^high^, MHC-II ^high^, Siglec-F negative, B220 negative, and Gr1 negative cells. Individual lung DC subsets were further gated as CD103^+^ DCs and CD11b^high^ DCs. Samples were acquired using a BD LSR-II flow cytometer at the Flow Cytometry Core Facility, Mount Sinai School of Medicine. Dead cells were excluded by DAPI staining. Data was analyzed using FlowJo software (Treestar Corp.)

MLNs and lungs were isolated from infected and control mice as described above under sterile conditions for cell sorting. Total cell suspensions or enriched DC fractions were used to sort individual CD103^+^ DC, CD11b^high^ DC, and CD8α^+^ DC subsets. Other MLN cell types such as B cells, pDCs and Gr1^+^ cells were sorted from total cell suspensions. CD11c^+^ cells were positively selected using anti-CD11c beads (Miltenyi Biotech) or by negative selection utilizing a cocktail of biotinylated mabs, that included anti-Gr1 (clone RB6-8C5), anti-CD19 (clone 6D5), anti-Ter119 (clone Ter-119), anti-CD3 (clone 17A2), and anti-CD49b (clone DX5) followed by incubation with anti-biotin beads (Miltenyi Biotec). In both cases, cells were passed through LS magnetic columns (Miltenyi Biotec) and positive or negative fractions were collected depending on the method employed, then cells were stained as described above and sorted using a BD Aria-II cell sorter (Flow Cytometry Core Facility, Mount Sinai School of Medicine).

### Immunohistochemistry of mediastinal lymph nodes

MLNs isolated from PR8 infected mice, were embedded in optimal cutting temperature (O.C.T) medium (Tissue-Tek), frozen over dry ice, and stored at -80° C until further use. 8 µm sections were cut with using a cryostat (Leica), placed over coated microscope slides, and fixed with 4% paraformaldehyde in PBS for 5 min at room temperature. Sections were blocked with PBS containing 2% FCS and 1% mouse serum for 20 min. Then, incubated with purified rat anti-B220 (clone RA3-6B2) and purified hamster anti-CD11c (clone HL3) for 45 min, washed twice with PBS and incubated with goat anti-rat Alexa-647 (Invitrogen) and goat-anti-hamster FITC (Jackson Immunoresearch) polyclonal sera, to visualize B220 (B cell areas) and CD11c (DCs), respectively. Following surface staining, slides were permeabilized with PBS containing Saponin 0.02% for 30 min and then incubated with a mixture of biotinylated antibodies to influenza NP (clone HT103) and M (2E10) proteins. Slides were washed twice and then incubated with Streptavidin-Cy3 (Sigma). Slides were air-dried, and mounted for fluorescence microscopy using Prolong Antifade with DAPI (Invitrogen). Immunofluorescence was performed using a Zeiss Axioplan2 fluorescence microscope. Images were analyzed using ImageJ software.

### Determination of lung and lymph node virus titers

Plaque immunostaining assays were performed to quantitate virus titers from infected mice. Lung homogenates from infected mice (1 lung was homogenized in 1.8 ml of PBS containing 0.1% Gelatin) were 10-fold serially diluted. 24 well plates were seeded with Mardy-Darby Canine Kidney (MDCK) cells, 1 day before the infection to achieve confluent monolayers. The plates were washed 3 times with DMEM, and incubated for 1 hr with 100 µl of infected-lung homogenates. After the inoculums were removed, the cells were washed once with DMEM, and 500 µl of overlay media (DMEM-F12 containing 0.6% agar (Oxoid), 0.5% Albumin (MP biomedicals), 0.1% NaHCO3, antibiotics and 1 µg/ml TPCK-trypsin (Worthington) ) was added on top. Infected monolayers were incubated for 40 h at 37°C, and then fixed with 4% paraformaldehyde for 1 hr. Agar overlays were removed gently under running water. Fixed monolayers were washed with PBS twice, and incubated with chicken anti-PR8 polyclonal sera (Charles River) followed by goat HRP-anti-chicken (Jackson Immunoresearch). Plaques were visualized after incubation with the True Blue substrate (KPL) that produces a blue precipitate by an HRP mediated reaction. Plaque forming units (pfu) were counted and virus titers were expressed as pfu/ml.

To determine virus titers in lymph nodes, MLNs were isolated at different time points after infection and homogenized in 500 µl of PBS. Immediately after homogenization, samples were pooled, and 10-fold serial dilutions were injected in triplicates into 10-day embryonated-chicken eggs. 40 h post-inoculation, allantoic fluid was harvested to determine virus particles by hemagglutination of red blood cells (RBCs). Endpoint titers were determined by the method of Reed and Muench [87].

### *In vitro* T cell proliferation assay

A CD8^+^ T cell isolation kit (Miltenyi Biotec) was used to isolate untouched OT-I CD8^+^ T cells following manufacturer instructions. OT-I naive T cells were labeled with carboxyfluorescein diacetate succinimidyl ester (CFSE) at a final concentration of 2.5 µM. Individual MLN CD11c^+^ cell populations (CD103^+^ DCs, CD11b^high^ DCs and CD8α^+^ DCs) were isolated at day 4 post-infection from wild type or IFNAR ^-/-^ mice infected with PR8-OT-I and co-cultured in 96 well plates with CFSE-labeled OT-I transgenic T cells at a ratio of 10,000 DCs to 50,000 CFSE-labeled transgenic T cells per well. 60 h later, T cell proliferation was quantitated by CFSE dilution using flow cytometry as described elsewhere [9]. Dead cells were excluded using PI or DAPI staining.

### Confocal microscopy of migratory DCs

A suspension of sorted CD103^+^ DC and CD11b^high^ DCs in 100 µl, were placed over microscope poly-lysine coated slides (Shandon) and let to adhere for 2 h at 37°C in an incubator inside a humidified hybridization chamber (Sigma) in order to avoid evaporation of the media. After the incubation time was over, the media was rapidly aspirated (avoiding the cells to dry), and a drop of ∼100 µl of freshly prepared 4% paraformaldehyde in PBS was added to fix the adherent cells at room temperature. After 3 min, the solution was aspirated and replaced with fresh paraformaldehyde 4% (100 µl per sample) and the cells were fixed for additional 15 min. Cells were permeabilized using a solution of Saponin 0.02% dissolved in PBS. Slides were blocked with PBS containing 1% FBS, 1% mouse serum (Jackson Immunoresearch) and Saponin 0.02% for 20 min, and stained for viral NP with biotinilated-HT103 monoclonal antibody for 30 min at room temperature. Followed by 3 washes in Saponin-PBS, secondary staining was performed using Streptavidin-Cy3 (Sigma).

Slides were air-dried and Prolong Antifade with DAPI (Invitrogen) was used as mounting media.

Fluorescence Microscopy was performed on a Zeiss Inverted LSM 510 laser scanning confocal instrument mounted on a Zeiss Axiovert 200M microscope. All images were acquired using a 100x oil objective. Images were analyzed with ImageJ software.

### Determination of infectious virus particle production by migratory DCs

MLN DCs from PR8 (day 2 to day 4 post-infection) or WSN infected mice (day 3 post-infection) were sorted by FACs and co-cultured for 3 days with confluent MDCK cells seeded on 96 well plates in DMEM media containing 0.5% Albumin (MP biomedicals), 0.1% NaHCO3 and antibiotics. The culture media was either supplemented or not with TPCK-trypsin (1 µg/ml). Supernatants from these cultures were assayed for the presence of replicating virus by hemagglutination of RBCs to determine whether DCs were capable of transferring infectious virus particles to MDCKs *in vitro*. Infected MDCK cells were visualized via immunostaining with polyclonal anti-chicken PR8 developed with horseradish peroxidase reaction with True Blue substrate as described for plaque immunostaining assays. Images of infected MDCK cells were acquired using a Nikon Eclipse TS100 microscope light microscopy. Alternatively, sorted DCs or other sorted cells from the MLNs were injected directly into 10-day embryonated eggs. 2 days later, allantoic fluid was isolated and presence of virus was determined by hemagglutination of RBCs. In parallel, allantoic fluid from these experiments was serially diluted and used to infect MDCK cells to determine whether the virus in these preparations was infectious as measured by plaque immunostaining on MDCK cells.

### Real-time quantitative PCR (qPCR)

Lungs or lymph nodes (3–5 pooled lymph nodes) from infected mice were homogenized in 3 ml/sample of Trizol Reagent (Invitrogen) and RNA was isolated as indicated by the manufacturer. Total mRNA was converted to cDNA by RT-PCR using oligo-dT reaction (Affinity Script, Stratagene). cDNA was diluted 50 times in water and triplicate reactions were setup in 384-well plates. qPCR reactions based on SYBR green detection, were performed using a Lightcycler equipment (Roche, USA) all reactions were normalized to α-tubulin as previously described [88].

qPCR reactions with sorted DCs required mRNA amplification. The WT-OVATION amplification Kit (Nugen, San Diego, USA) was used for this purpose as described by the supplier.

The list of primers used in this study is the following:

M1/2 forward: 5′-GGGAAGAACACCGATCTTGA-3′; M1/2 reverse: 5′-CGGTGAGCGTGAACACAAAT-3′; NA forward: 5′-CATCTCTTTGTCCCATCCGT-3′; NA reverse: 5′-GTCCTGCATTCCAAGTGAGA-3′; HA forward: 5′-GAGGAGCTGAGGGAGCAAT-3′; HA reverse: 5′-GCCGTTACTCCGTTTGTGTT-3′; PB1 forward: 5′-CCTCCTTACAGCCATGGGA-3′; PB1 reverse: 5′-GTGCTCCAGTTTCGGTGTTT-3′; PB2 forward: 5′-GGATCAGACCGAGTGATGGT-3′; PB2 reverse: 5′-CCATGCTTTAGCCTTTCGACT-3′; PA forward: 5′-CATCAATGAGCAAGGCGAGT-3′; PA forward: 5′-GCCCCTGTAGTGTTGCAAAT-3′; NP forward: 5′-CAGCCTAATCAGACCAAATG-3′; NP reverse: 5′-TACCTGCTTCTCAGTTCAAG-3′;NS1 forward: 5′-TTCACCATTGCCTTCTCTTC-3′;

NS1 reverse: 5′-CCCATTCTCATTACTGCTTC-3′; *Mx1* forward: 5′-CAACTGGAATCCTCCTGGAA-3′; *Mx1* reverse: 5′-GGCTCTCCTCAGAGGTATCA-3′; *Isg15* forward: 5′-GAGCTAGAGCCTGCAGCAAT-3′; *Isg15* reverse: 5′-CTTCTGGGCAATCTGCTTCT-3′; *Bst2* forward: 5′- CAAACTCCTGCAACCTGACC-3′;

*Bst2* reverse: 5′- CATTCTCAAGCTCCTTGATGC-3′; *Ifnar1* forward: 5′ -GGTTGATCCGTTTATTCCATTC-3′; *Ifnar1* reverse: 5′- CCACATGTTCCCGTCTTGT-3′;

*Ifnar2* forward: 5′-CTTCGTGTTTGGTAGTGATGGT-3′; *Ifnar2* reverse: 5′-GGGGATGATTTCCAGCCGA -3′; *Stat1* forward: 5′-CTTCAGCAGCTGGACTCCAA -3′; *Stat1* reverse: 5′- GGTCGCAAACGAGACATCAT-3′; *Stat2* forward: 5′-AAGAGGTGCAGCCCCCACCA-3′; *Stat2* reverse: 5′-GCTGCGCCTGTTGGCTCTGA-3′; *Jak1* forward: 5′- TGCAGGAGGGAGCCTGGCAT-3′; *Jak1* reverse: 5′-AGCTTGCCCCAGGGGATCGT-3′; *Tyk2* forward: 5′-AGCCATCTTGGAAGACAGCAA-3′; *Tyk2* reverse: 5′-GACTTTGTGTGCGATGTGGAT-3′; α-tubulin forward: 5′-TGCCTTTGTGCACTGGTATG-3′; α-tubulin reverse: 5′-CTGGAGCAGTTTGACGACAC-3′.

### Lung DC migration blockade by pertussis toxin (PTX) during infection

2 h after aerosol infection, mice were anesthetized and 2 µg of PTX dissolved in 35 µl of PBS was delivered intranasally to each mouse. Once a day thereafter, mice received 0.5 µg of PTX i.p to maintain the chemokine receptor blockade. MLNs and lungs of mock and PTX treated mice were collected at day 1, 2, and 3 post-infection. To further confirm ablation of lung DC migration, absolute numbers of CCR7^+^ CD11c^+^ cells were determined in infected mice treated with PBS or PTX. Mouse anti-CCR7 antibody (clone 4B12) was used for this experiment.

### Diphtheria toxin depletion of DCs during infection

Langerin^+^ CD103^+^ DCs were depleted in PR8-infected Langerin-DTR EGFP mice with an intraperitoneal (i.p) injection of 1 µg of diphtheria toxin (DT, Sigma), 1 day before infection. At day 0, mice were infected with aerosolized PR8 virus. Thereafter, a daily dose of 200 ng was administered i.p to each mouse to maintain the DC depletion. Lungs and MLNs were harvested every day for RNA extraction and virus titer determination. For total CD11c^+^ cell depletion, CD11c-DTR EGFP mice were injected i.p with 4 ng/g (body weight) of DT, 1 day before PR8 infection. Thereafter daily doses of 30 ng of DT were administered i.p up to day 3. Viral message in the MLNs was determined by qPCR as described above. Lung titers were determined by plaque immunostaining assay.

### Cytokine ELISA

To determine the concentration of IFN-γ from mixed DC-T cell cultures, an ELISA kit from R&D (UK) was used according to the manufacturer's instructions.

### Statistical analysis

Averaged results were expressed as means+/- standard deviation. A two-tailed Student's t test was used to determine statistical significance of selected samples. P values< 0.05 (95% Confidence) were considered to be significant. Graphs were designed either in Excel or Graph Pad software.

### Gene accession list

CD11c (name: Itgax, ID: 16411); CD11b (name: Itgam, ID: 16409); CD103 (name: Itgae, ID: 16407); *Ifnar1* (name: Ifnar1, ID: 15975); *Ifnar2* (name: Ifnar2, ID: 15976); *Stat1* (name: Stat1, ID: 20846); *Stat2*(name: Stat2, ID: 288774); *Jak1*(name: Jak1, ID: 16451); *Tyk2* (name: Tyk2, ID: 54721); Ly6C (name: Ly6c1, ID: 17067); Ly6G (name: Ly6g, ID: ); CD8α (name: Cd8a, ID: 12525); *Isg15* (name: Isg15, ID: 100038882); *Mx1* (name: Mx1, ID: 17857); *Bst2* (name: Bst2, ID: 69550); *IFN-γ* (name: Ifng, ID: 15978); Siglec-F (name: Siglec5, ID: 233186); B220 (name: Ptprc, ID: 19264); CD45 (name: Ptprc, ID: 19264); Langerin (name: Cd207, ID: 246278); CCR7 (name: Ccr7, ID: 12775); HA (name: HA, ID: 956529); NP (name: NP, ID: 956531); NS1 (name: NS1, ID: 956533); NA (name: NA, ID: 956530); PA (name: PA, ID: 956535); PB1 (name: PB1, ID: 956534); PB2 (name: PB2, ID: 956536); M ((name: M1, ID: 956527 and name: M2, ID: 956528 ); MHC-II (name: H2-Ab1, ID: 14961 and name: H2-Aa, ID: 14960).

## Supporting Information

Figure S1 **Migratory DCs can transfer infectious virus to uninfected cells in the absence of TPCK-trypsin.** A. MLN-DCs (gate V, figure 3B) from PR8 or WSN infected mice (day 3) were co-cultured with MDCK cells in the presence or absence of TPCK-trypsin for 2 days. Virus replication was assessed by HRP-based immunostaining of MDCK cells with a chicken polyclonal antibody to influenza virus. Control wells were stained with chicken normal serum followed by HRP-based immunostaining. B. Sorted CD103^+^ DCs and CD11b^high^ DCs from PR8 or WSN-infected mice were co-cultured with MDCK cells in the presence or absence of TPCK-trypsin and the culture supernatants were assayed for infectious virus particles at day 2 by hemmaglutination of RBCs.
(PDF)

Figure S2 **Sorting strategy to isolate LN-resident CD8α^+^ DCs during influenza virus infection.** LN-resident CD8α^+^ DCs were sorted by gating progressively through gates I-V to eliminate contaminant lymph-node cells. From gate V, CD11c^high^ MHC-II ^intermediate^ DCs were further separated for cells that express high levels of CD8α (gate VII), denoted in the manuscript as LN-resident CD8α^+^ DCs. From gate VI, CD11c^high^ MHC-II^high^ cells were separated as previously shown in Figure 3, for CD103^+^ DCs (gate VIII) and CD11b^high^ DCs (gate IX).
(PDF)
