# Supplementary material for: Unique Type I Interferon Responses Determine the Functional Fate of Migratory Lung Dendritic Cells during Influenza Virus Infection
Source: PLoS Pathog. 2011 Nov 3;7(11):e1002345. doi: 10.1371/journal.ppat.1002345 (PMC3207893; doi:10.1371/journal.ppat.1002345)
Supplement: Figure S1 — Migratory DCs can transfer infectious virus to uninfected cells in the absence of TPCK-trypsin. A. MLN-DCs (gate V, figure 3B) from PR8 or WSN infected mice (day 3) were co-cultured with MDCK cells in the presence or absence of TPCK-trypsin for 2 days. Virus replication was assessed by HRP-based immunostaining of MDCK cells with a chicken polyclonal antibody to influenza virus. Control wells were stained with chicken normal serum followed by HRP-based immunostaining. B. Sorted CD103+ DCs and CD11bhigh DCs from PR8 or WSN-infected mice were co-cultured with MDCK cells in the presence or absence of TPCK-trypsin and the culture supernatants were assayed for infectious virus particles at day 2 by hemmaglutination of RBCs. (PDF) [file ppat.1002345.s001.pdf]

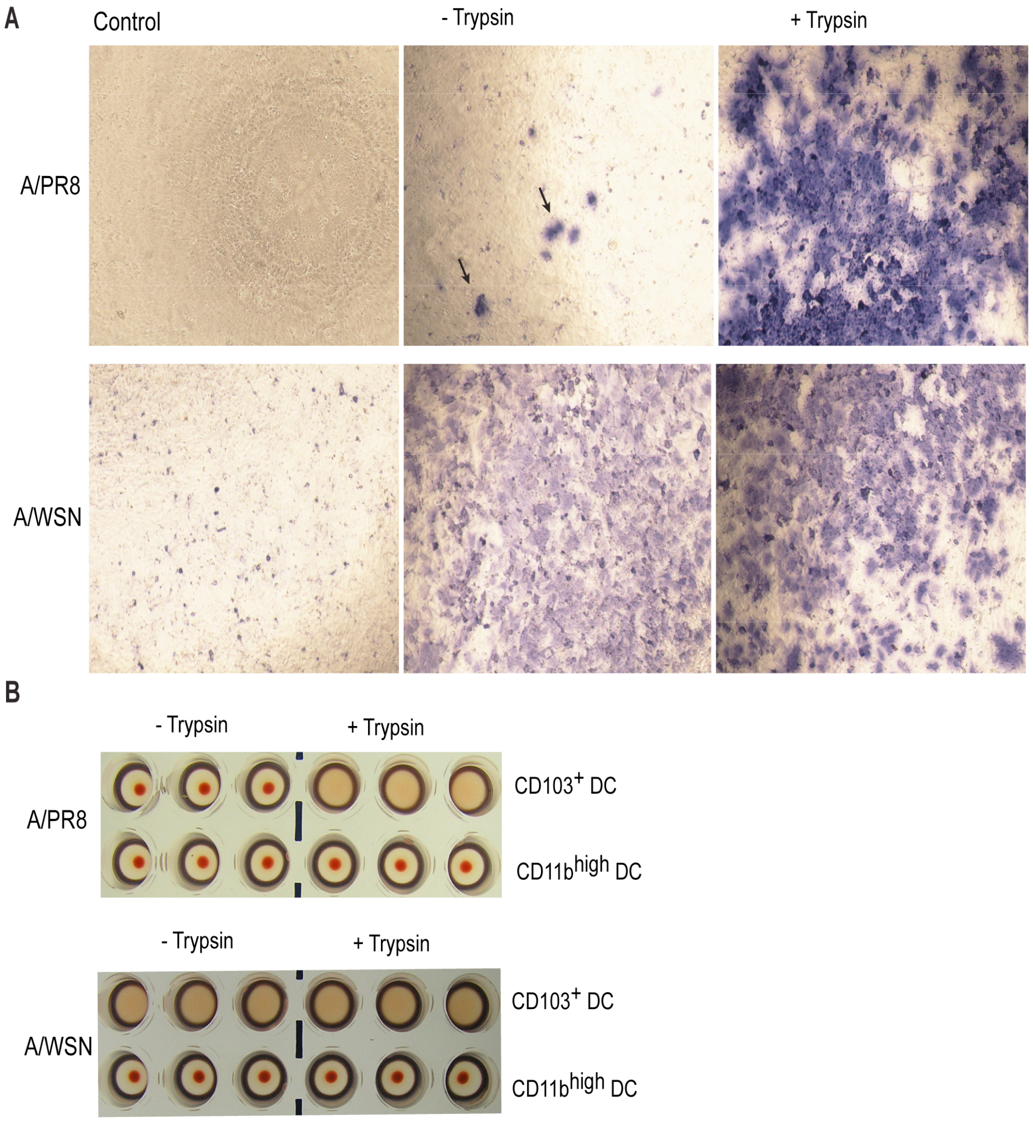

Figure S1. Migratory DCs can transfer infectious virus to uninfected cells in the absence of TPCK-trypsin A. MLN-DCs (gate V, figure 3B) from PR8 or WSN infected mice (day 3) were co-cultured with MDCK cells in the presence or absence of TPCK-trypsin for 2 days. Virus replication was assessed by HRP-based immunostaining of MDKC cells with a chicken polyclonal antibody to influenza virus. Control wells were stained with chicken normal serum followed by HRP-based immunostaining. B. Sorted CD103^{+} DCs and CD11b^{high} DCs from PR8 or WSN-infected mice were co-cultured with MDCK cells in the presence or absence of TPCK-trypsin and the culture supernatants were assayed for infectious virus particles at day 2 by hemmaglutination of RBCs.
