# Supplementary material for: Unique Type I Interferon Responses Determine the Functional Fate of Migratory Lung Dendritic Cells during Influenza Virus Infection
Source: PLoS Pathog. 2011 Nov 3;7(11):e1002345. doi: 10.1371/journal.ppat.1002345 (PMC3207893; doi:10.1371/journal.ppat.1002345)
Supplement: Figure S2 — Sorting strategy to isolate LN-resident CD8α+ DCs during influenza virus infection. LN-resident CD8α+ DCs were sorted by gating progressively through gates I-V to eliminate contaminant lymph-node cells. From gate V, CD11chigh MHC-II intermediate DCs were further separated for cells that express high levels of CD8α (gate VII), denoted in the manuscript as LN-resident CD8α+ DCs. From gate VI, CD11chigh MHC-IIhigh cells were separated as previously shown in Figure 3, for CD103+ DCs (gate VIII) and CD11bhigh DCs (gate IX). (PDF) [file ppat.1002345.s002.pdf]

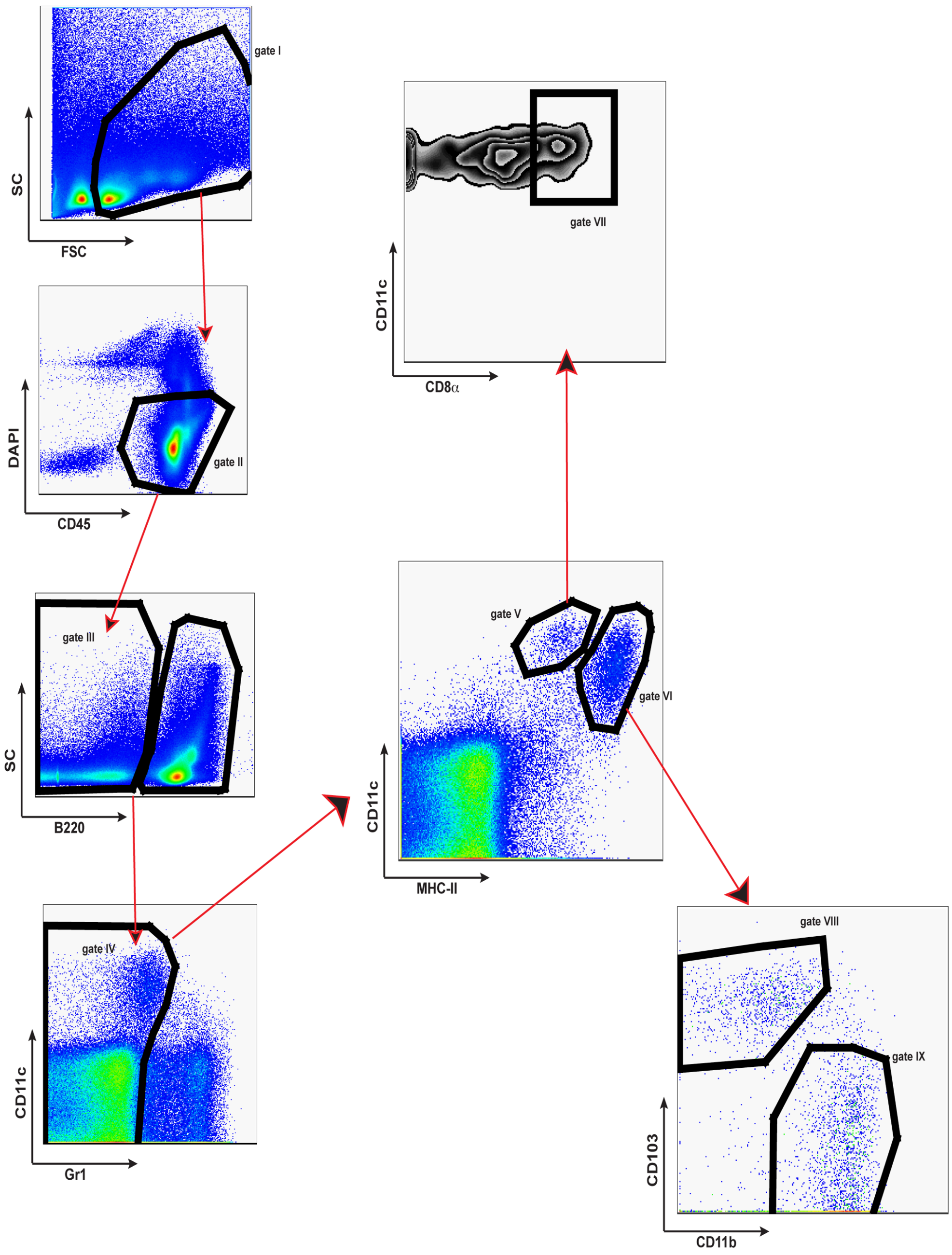

Figure S2. Sorting strategy to isolate LN-resident CD8α^{+} DCs during influenza virus infection. LN-resident CD8α^{+} DCs were sorted by gating progressively through gates I-V to eliminate contaminant lymph-node cells. From gate V, CD11c^{high} MHC-II ^{intermediate} DCs were further separated for cells that express high levels of CD8α (gate VII), denoted in the manuscript as LN-resident CD8α^{+} DCs. From gate VI, CD11c^{high} MHC-II^{high} cells were separated as previously shown in Figure 3, for CD103^{+} DCs (gate VIII) and CD11b^{high} DCs (gate IX).
